# Design and Analysis of a Flexible Smart Apparel MIMO Antenna for Bio-Healthcare Applications

**DOI:** 10.3390/mi13111919

**Published:** 2022-11-06

**Authors:** Thennarasi Govindan, Sandeep Kumar Palaniswamy, Malathi Kanagasabai, Sachin Kumar, Mohamed Marey, Hala Mostafa

**Affiliations:** 1 Department of Electronics and Communication Engineering, SRM Institute of Science and Technology, Kattankulathur 603203, India; 2 Department of Electronics and Communication Engineering, College of Engineering, Anna University, Chennai 600025, India; 3 Smart Systems Engineering Laboratory, College of Engineering, Prince Sultan University, Riyadh 11586, Saudi Arabia; 4 Department of Information Technology, College of Computer and Information Sciences, Princess Nourah bint Abdulrahman University, P.O. Box 84428, Riyadh 11671, Saudi Arabia

**Keywords:** flexible, MIMO antenna, smart apparel, smart textile, UWB, wearable

## Abstract

This paper presents the design and development of a quad-port smart textile antenna for bio-healthcare applications. The antenna is designed to operate in the ultra-wideband (UWB) spectrum (3.1–12 GHz) with an impedance bandwidth of 8.9 GHz. The size of the unit cell and multiple-input multiple-output (MIMO) antenna are 0.25$\lambda_{0}$ × 0.2$\lambda_{0}$ × 0.015$\lambda_{0}$ and 0.52$\lambda_{0}$ × 0.52$\lambda_{0}$ × 0.015$\lambda_{0}$, respectively. The antenna has a maximum efficiency of 93% and a peak gain of 4.62 dBi. The investigation of diversity metrics is performed and the results obtained are found to be ECC < 0.08 and DG < 9.99 dB. The computed CCL and TARC values are <0.13 bits/s/Hz and <−12 dB, respectively. The SAR analysis of the antenna shows a value of 0.471 Watt/Kg at 4 GHz, 0.39 Watt/Kg at 7 GHz, and 0.22 Watt/Kg at 10 GHz.

## 1. Introduction

Wearable electronics is a rapidly growing field that has piqued consumer interest in recent years. It has a wide range of applications in sports, military, space, mobile medical monitoring, and healthcare [1,2]. Wearable antennas are enormously useful in the medical field, for example by integrating them into garments, patients’ health data such as blood pressure, heart rate, metabolism, and body temperature can be obtained [3,4]. Smart textile antennas are becoming more popular due to their low cost, flexibility, and ease of production. They are comfortable to wear, even when bent [5]. Textile antennas for on-body applications should be lightweight and flexible. Some of the flexible materials used in smart clothing include jeans, cotton, polyester, denim, silk, felt, plastics, paper, and Tencel fabric [6,7]. An e-textile antenna was designed to establish data communication between a smartphone and a Bluetooth receiver for body area network applications [8]. In [9], a dual-band EBG integrated monopole antenna with fractal geometry was designed, and a 3 × 3 array was introduced on the backside of the antenna to reduce the specific absorption rate (SAR). In [10], a polygon-shaped wearable antenna was reported for the 900 MHz and 2400 MHz bands. The antenna was crumpled, bent, and tested for on-body performance. In [11], a breathable textile antenna operating at 2.45 GHz was presented. The antenna exhibited flexibility, and a 3D spacer substrate was used to allow the antenna to breathe. A jean-based textile antenna was designed in [12] for use in wireless communication applications. The antenna operates at three different frequencies: 2.13 GHz, 4.75 GHz, and 11.495 GHz, making it useful for emergency and monitoring applications.

The antennas in [13,14] used e-thread and conductive thread as materials. In [15], a low-cost carbon conductive ink was used on a cotton substrate. In [16], a textile antenna was used as a moisture sensor. The major drawback of textile materials is their limited bandwidth, which slows data transmission. In [17], an antenna with an E-shaped slot was reported to increase bandwidth. It is challenging to design a wideband textile antenna that is durable and transmits data consistently even when the human body’s posture changes. Multiple-input multiple-output (MIMO) antennas could be a solution to such problems [18]. In MIMO antennas, the mutual coupling should be minimized by keeping an adequate distance between the antenna elements. The goal of MIMO antenna designers is to develop a compact antenna with high inter-element isolation. In [19], an artificial magnetic conductor (AMC) was used to achieve high isolation. In [20], the electromagnetic band gap (EBG) structure was used to achieve high gain and in mitigating the harmful effects of radiation on the human body. The periodic (AMC/EBG) structures offer wider bandwidth and reduce unwanted surface waves. However, the addition of periodic structures increases the design complexity of the antenna.

This paper presents the design of an ultra-wideband (UWB) quad-port smart apparel wearable antenna for bio-healthcare applications. The term “smart” refers to the integration of antennas into the textile material, which allows it to be used as smart apparel to send sportspersons’ health conditions, energy metabolism, heart rate, etc. The term “smart” is coined as a result of the transformation of commonly available textile materials into a data-transceiving device such as an antenna for effective communication. The proposed antenna is developed on a polyester substrate to allow easy integration into clothing. The performance of the antenna is validated through surface current distribution plots. The unit antenna element is developed into a four-port MIMO antenna that can provide high-speed reliable data transmission. The diversity characteristics of the MIMO antenna are computed. The bending analysis is carried out in moderate and severe bending conditions. The SAR analysis of the antenna is also performed to investigate its effect on the human body. The proposed antenna could be useful in sports and healthcare applications owing to its flexibility and wrinkle-resistant behavior.

## 2. Antenna Design

### 2.1. Unit Cell Design

**Figure 1:**
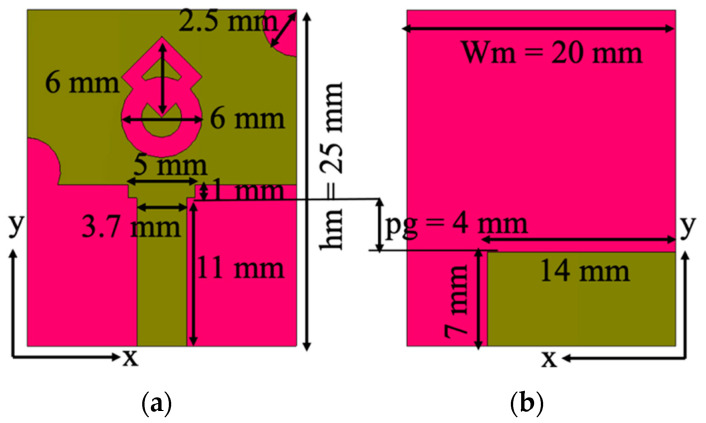
Antenna design: (**a**) front side, (**b**) back side.

Figure 1 displays the unit cell layout of the proposed antenna. The unit cell is designed on the polyester substrate with dielectric constant and loss tangents of 1.9 and 0.045, respectively [21]. Polyester is chosen as the substrate material because of its durability, lightweight, wrinkle resistance, strain resistance, and shape retention properties. The unit cell occupies a volume of 25 mm × 20 mm × 1.5 mm (0.25$\lambda_{0}$ × 0.2$\lambda_{0}$ × 0.015$\lambda_{0}$), where $\lambda_{0}$ is equivalent to the lowest operating frequency. The proposed antenna operates in the UWB range (3.1–12 GHz), and a partial ground plane is used to achieve better impedance matching.

The lowest operating frequency (*f_low_*) of a UWB monopole antenna is calculated using Equation (1) [22,23]

(1) $$\;f_{low}=\frac{7.2}{\left(h_{m}+w_{m}+p_{g}\right)\times k}\;$$

where $h_{m}$ is the height of the monopole antenna, $w_{m}$ is its width, and $p_{g}$ is the distance between the monopole radiator and the ground plane, which is 0.4 cm. Here, *k* is calculated by taking the root of the effective dielectric constant, and the Equation (1) is rewritten as

(2) $$f_{low}=\frac{7.2}{\left(1.217\pi \left[\left(r_{m}+l_{m}\right)\right]+p_{g}\right)\times k}\;$$

The expression ($h_{m}+w_{m}$) is rewritten as 1.217π($r_{m}+l_{m})$, which is the perimeter of a rectangular radiator. The terms $r_{m}$ and $l_{m}$ refer to the semi-width and semi-length of the monopole antenna.

**Figure 2:**
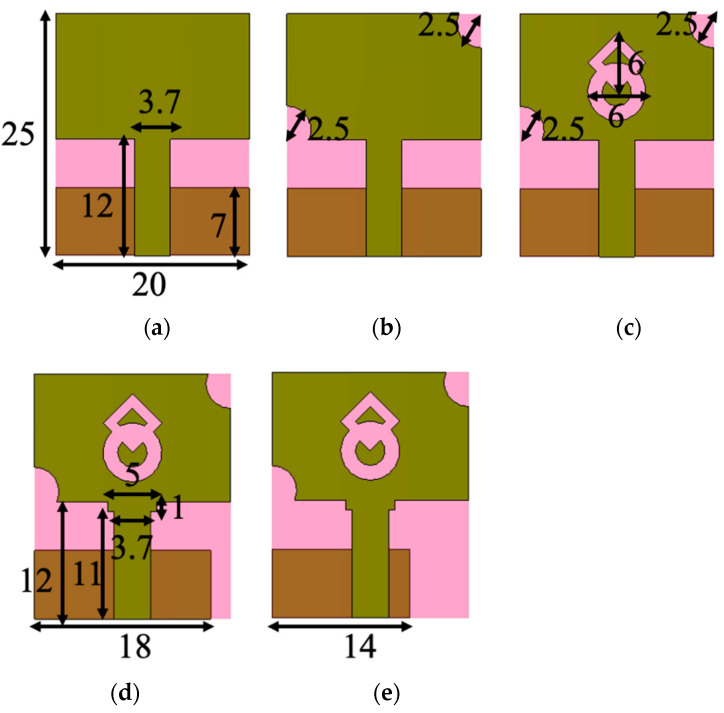
Evolution stages of the proposed antenna. (**a**) Evolution-1, (**b**) Evolution-2, (**c**) Evolution-3, (**d**) Evolution-4, and (**e**) Evolution-5.

**Figure 3:**
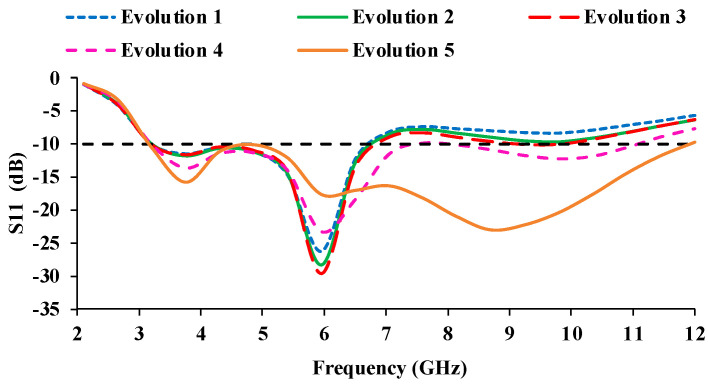
S_11_ (dB) curves of the evolution stages.

Figure 2a portrays a rectangular radiator with a modified ground plane (Evolution 1). In the next stage (Evolution 2), a combination of circular and square rings is loaded in the center of the radiator (Figure 2b). In the third stage (Evolution 3), the corners of the radiator are truncated with symmetric semi-circular slots, which improves the impedance matching. It has a frequency range of 3.1–6.5 GHz (with an impedance bandwidth of 3.4 GHz) and 9–9.8 GHz. In the fourth stage (Evolution 4), the ground plane size is reduced by 2 mm from the top edge, and 4 mm from the side edge near the feedline, as shown in Figure 2d, resulting in an impedance bandwidth coverage of 3.1–7.1 GHz and 8.1–10.8 GHz. Additionally, the feedline is modified to a step-shaped geometry to improve impedance matching. The ground plane is further truncated (6 mm) from the edge to increase operational bandwidth (Evolution 5), as shown in Figure 2e, resulting in a wider bandwidth of 8.9 GHz (3.1–12 GHz). Figure 3 shows the reflection coefficient curves of the evolution stages. The antenna operates at frequencies ranging from 3.1 to 12 GHz.

**Figure 4:**
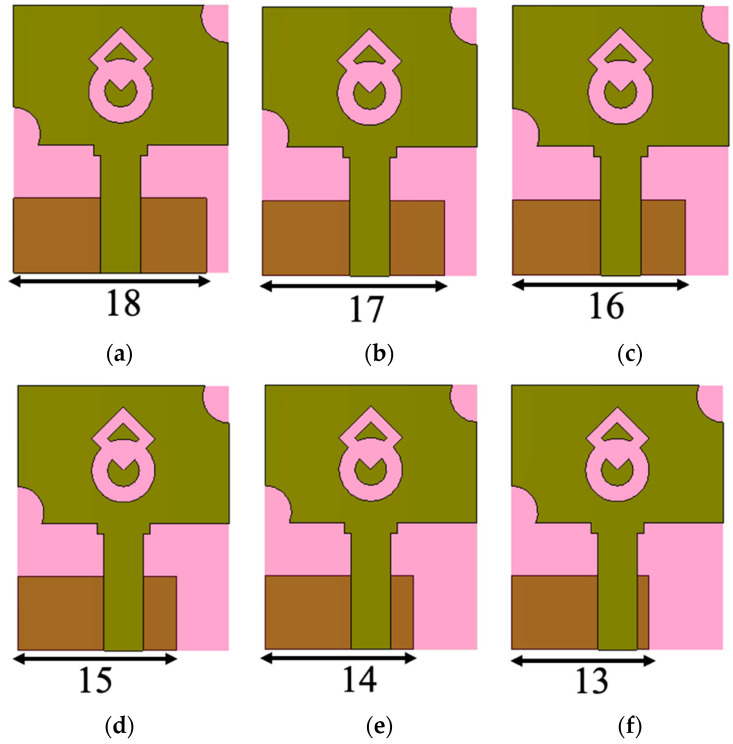
Parametric analysis of the antenna with varying ground plane width (GW). (**a**) GW = 18 mm, (**b**) GW = 17 mm, (**c**) GW = 16 mm, (**d**) GW = 15 mm, (**e**) 14 mm, (**f**) 13 mm.

**Figure 5:**
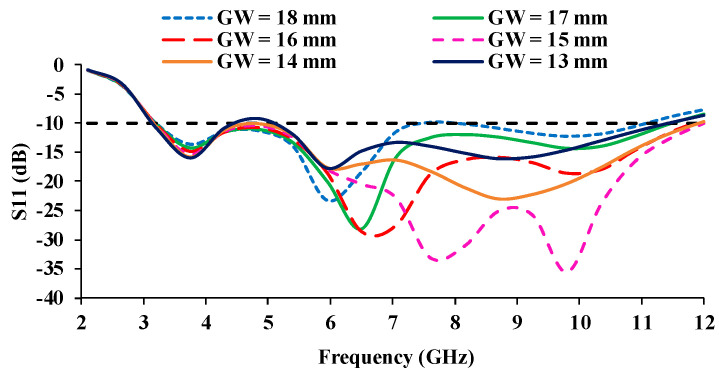
S_11_ (dB) curves for different ground plane width (GW).

The performance analysis is carried out by varying the ground plane width (GW) from 18 mm to 13 mm, as shown in Figure 4, and the corresponding S-parameters are shown in Figure 5. At GW = 18 mm, the entire UWB range is not covered, as there is some impedance mismatch in the frequency range of 7.3–8.2 GHz. When the GW is reduced to 17 mm, the proposed antenna covers UWB frequencies from 3.1 GHz to 10.6 GHz with an impedance bandwidth of 7.5 GHz. When the GW is reduced further, from GW = 16 mm to 14 mm, the impedance bandwidth on the higher frequency side increases, resulting in a larger impedance bandwidth of 8.9 GHz (3.1–12 GHz). When the ground plane width is reduced to 13 mm, the impedance begins to degrade in the 4.2–5.3 GHz frequency range. Therefore, the ground plane truncation is kept as 14 mm to achieve an impedance bandwidth of 8.9 GHz.

**Figure 6:**
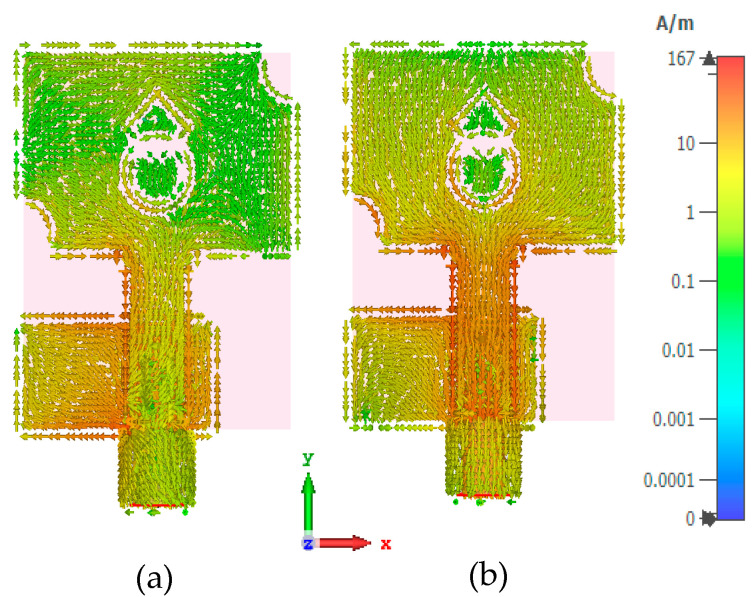
Current distribution at (**a**) 4 GHz, (**b**) 10 GHz.

**Figure 7:**
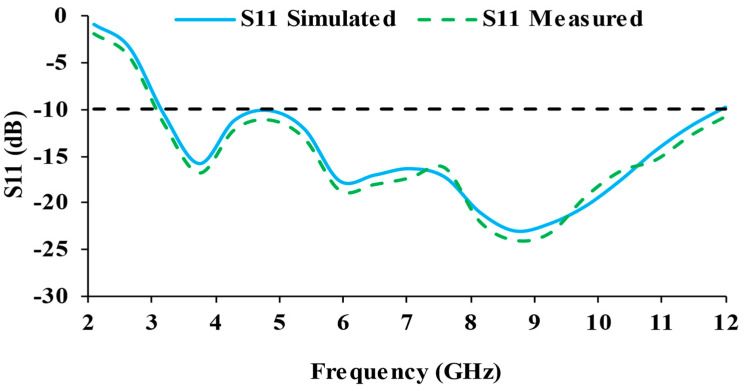
S_11_ (dB) curves of the designed antenna.

For a better knowledge of radiation performance, the current distribution of the antenna is analyzed. Figure 6 depicts the current distribution of the antenna. The current is highly distributed in the ground plane at 4 GHz. At 10 GHz, the current distribution is more concentrated in the feedline and close to the semi-circular slot. Figure 7 presents the reflection coefficient (simulated and measured) plots of the designed antenna. The simulated impedance bandwidth ranges from 3.1 to 12 GHz, while the measured bandwidth ranges from 3.07 to 12 GHz.

### 2.2. MIMO Antenna Design

**Figure 8:**
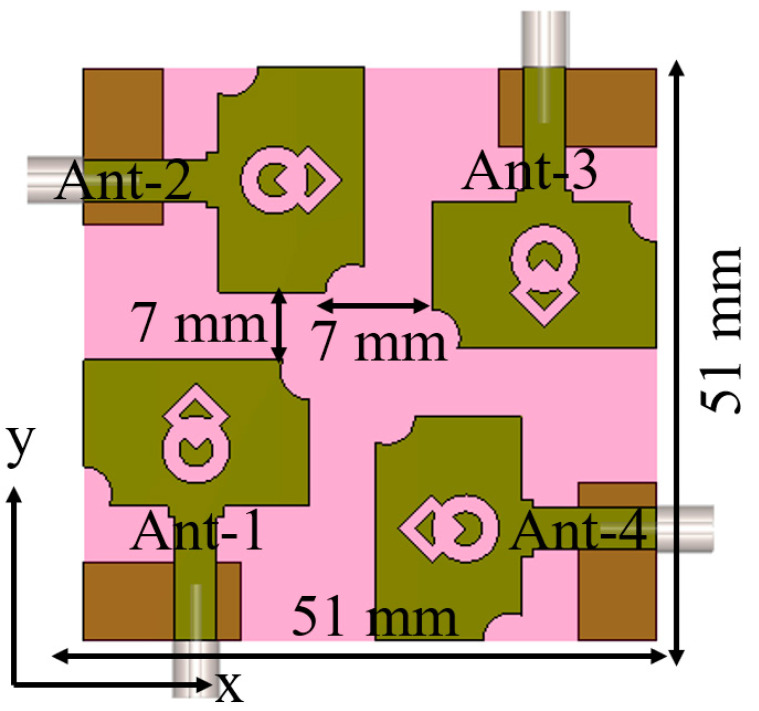
MIMO antenna design.

**Figure 9:**
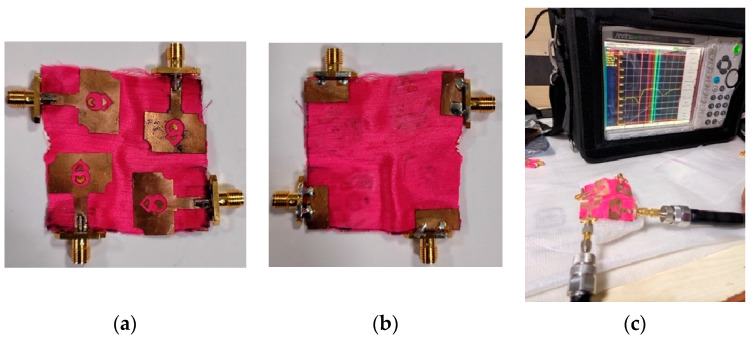
Fabricated MIMO antenna: (**a**) front side, (**b**) back side, (**c**) antenna measurement using VNA.

The developed MIMO antenna layout is displayed in Figure 8, where four-unit cells are duplicated and arranged in an orthogonal pattern. The spacing between the antenna elements is kept as 0.97$\lambda_{0}$. The MIMO antenna occupies a volume of 51 mm × 51 mm × 1.5 mm (0.52$\lambda_{0}$ × 0.52$\lambda_{0}$ × 0.015$\lambda_{0}$), where $\lambda_{0}$ is the wavelength corresponding to the lowest operating frequency. The prototype is fabricated on the polyester substrate, as depicted in Figure 9, and the S-parameter results are measured using Anritsu MS203C Vector Network Analyzer (VNA).

## 3. Discussion of the Findings

### 3.1. Scattering Parameters

**Figure 10:**
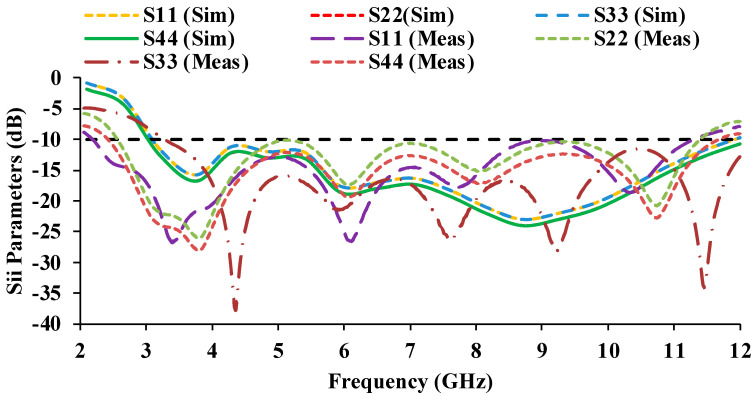
Simulated and measured reflection coefficient plots of the MIMO antenna.

**Figure 11:**
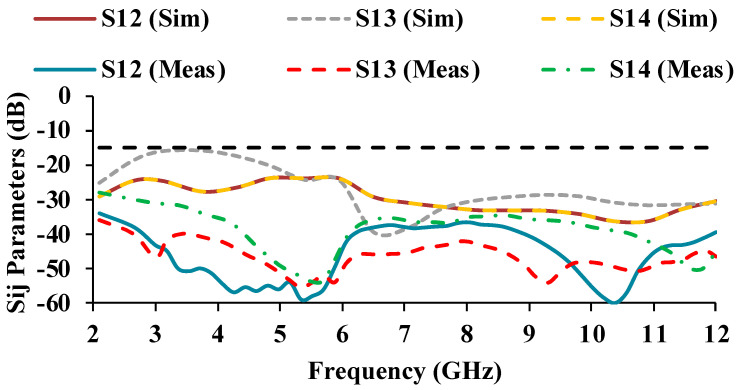
Simulated and measured mutual coupling plots of the MIMO antenna.

The simulated and measured scattering parameter results are displayed in Figure 10 and Figure 11. It can be noticed that the reflection coefficients of Antenna-1 and Antenna-3 are identical, and the reflection coefficients of Antenna-2 and Antenna-4 are also matching, which is due to the orthogonal placement of the unit cells. The simulated S-parameter has an impedance bandwidth of 8.9 GHz (3.1–12 GHz), while the measured S-parameter has an impedance bandwidth of 9.08 GHz (2.2–11.28 GHz). The simulated and measured results both cover the entire UWB range.

The mutual coupling characteristics of the measured results are lower than the simulated results. The mutual coupling may be reduced as a result of environmental changes [24,25]. In the proposed antenna, an isolation of greater than 17 dB is obtained over the entire UWB range.

### 3.2. Radiation Characteristics

The designed MIMO antenna is tested in an anechoic chamber with a standard horn antenna as the reference antenna on the transmitter side and the proposed antenna as the test antenna on the receiver side. The radiation pattern and gain are measured in an anechoic chamber. When port-1 is excited, the remaining ports (2, 3, and 4) are terminated with 50-ohm impedance, and vice versa.

The Friis transmission equation is used to calculate the gain

(3) $$\frac{P_{r}}{P_{t}}=G_{t}G_{r\;}{\left(\frac{\lambda }{4\pi d}\right)}^{2}$$

where

$P_{t}$ is the power delivered by the transmitting antenna, and $P_{r}$ is the power available at the receiving antenna.
$G_{t}$ is the transmitter antenna gain, and $G_{r}$ is the receiver antenna gain.
λ is the wavelength, and d is the distance between the transmitter and receiver.

The directivity is calculated using Equation (4) [26]

(4) $$D=\frac{41,253}{\theta_{E\;}\theta_{H}}$$

where $\theta_{E}$ and $\theta_{H}$ are the half-power beam widths obtained in the *E* and *H* planes, respectively.

The measured radiation efficiency is calculated using Equation (5)

(5) $$\eta =\frac{G}{D}$$

where G is the gain, D is the directivity, and η is the efficiency of the antenna.

**Figure 12:**
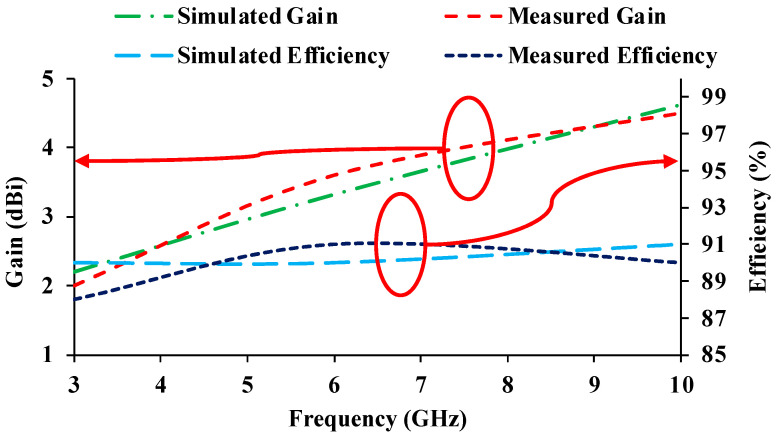
Gain and efficiency curves of the antenna.

**Figure 13:**
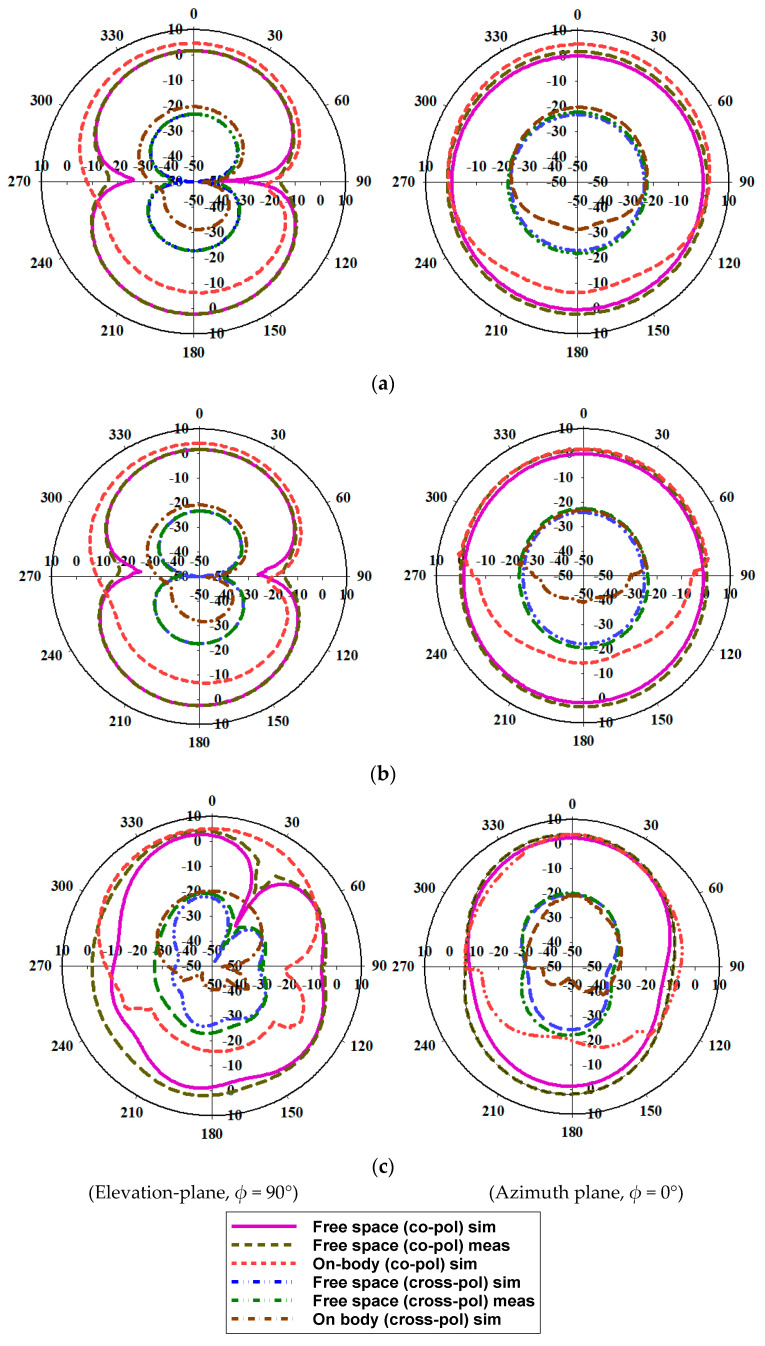
Radiation patterns of the antenna (gain in dBi and angle in degree). (**a**) 4 GHz; (**b**) 7 GHz; (**c**) 10 GHz.

Figure 12 presents the simulated and measured gain and efficiency plots of the MIMO antenna. The simulated gain at 3 GHz, 6 GHz, and 10 GHz is 2.2 dBi, 3.32 dBi, and 4.82 dBi, respectively. Whereas, the measured gain values are 2 dBi, 4 dBi, and 4.7 dBi at 3 GHz, 6 GHz, and 10 GHz, respectively. The proposed antenna has a maximum efficiency of 92%. The radiation patterns can be seen in E-plane (elevation plane) and H-plane (azimuth plane) as depicted in Figure 13. When the polarization of the proposed antenna and the reference (Horn) antenna are the same, the co-polarization pattern is depicted. When the polarization of the antennas differs, a cross-polarization pattern is observed. The simulation results are plotted for the free-space and on-body conditions. The designed antenna is fabricated and measured in free space. When compared to the free space results, the on-body radiation patterns are suppressed due to the effects of the human body.

## 4. Diversity Performance

**Figure 14:**
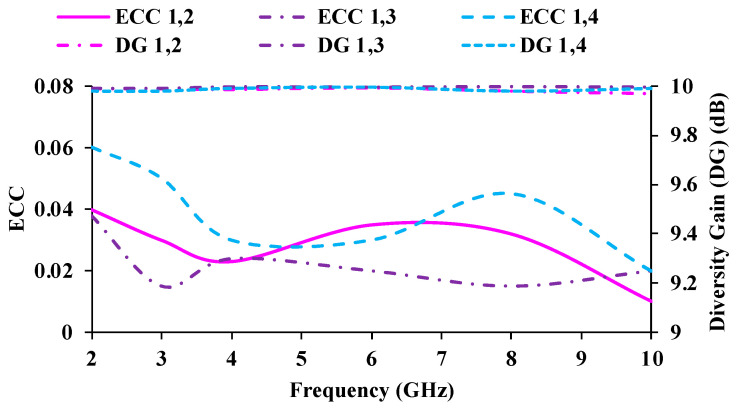
ECC and diversity gain plots of the MIMO antenna.

The diversity metrics of the developed antenna are investigated for far-field conditions. The calculated envelope correlation coefficient (ECC) and diversity gain (DG) of the MIMO antenna are depicted in Figure 14. ECC denotes the closeness of adjacent antenna unit cells [27], which can be evaluated using Equation (6). The practical limit of the ECC is less than 0.5. The ECC value in Figure 14 shows a low correlation between two neighboring antenna elements due to the adequate spacing of 7 mm (0.97*λ*_0_) between them. The spacing between the antenna elements results in a good isolation of >17 dB. As a result, the ECC plots show low correlation values.

(6) $$ECC=\frac{{\left|\iint \left[\vec{F_{1}}\left(\theta ,\varphi \right)\cdot \vec{F_{2}}\left(\theta ,\varphi \right)\right]d\Omega \right|}^{2}}{\iint {\left|\vec{F_{1}}\left(\theta ,\varphi \right)\right|}^{2}d\Omega \;\iint {\left|\vec{F_{2}}\left(\theta ,\varphi \right)\right|}^{2\;}d\Omega }$$

where $F_{1}$ and $F_{2}$ are the radiated fields and the notations θ, φ are the elevation (vary from 0 to π) and azimuth angles (vary from 0 to 2π). The phase values of the radiation patterns are determined by using angles (θ,φ). Equation (6) is derived from Equation (7).

(7) $$ECC_{i,\;j}={\left|\frac{\int_{0}^{\pi }\int_{0}^{2\pi }\left(XPR\cdot \;E_{\theta i}\cdot E_{\theta j}^{*}\cdot \;P_{\theta }+XPR\cdot \;E_{\varphi i}\cdot E_{\varphi j}^{*}\cdot \;P_{\varphi }\right)\;\sin\left(\theta \right)\;d\theta \;d\varphi }{\sqrt{\prod_{k=i,j}\int_{0}^{\pi }\int_{0}^{2\pi }\left(XPR\cdot \;E_{\theta i}\cdot E_{\theta j}^{*}\cdot \;P_{\theta }+XPR\cdot \;E_{\varphi i}\cdot E_{\varphi j}^{*}\cdot \;P_{\varphi }\right)\;\sin\left(\theta \right)\;d\theta \;d\varphi }}\right|}^{2}\;$$

where *XPR* denotes the cross-polarization ratio between vertical (*P_V_*) and horizontal power (*P_H_*) components. The variables *i* and *j* represent port numbers. The field components in the elevation and azimuth directions are denoted by $E_{\theta i},E_{\theta j}^{*},E_{\varphi i},E_{\varphi j}^{*}.$ The power distribution in the elevation and azimuthal directions is denoted by $P_{\theta }$ and $P_{\varphi }$.

The terms $\int_{0}^{\pi }\int_{0}^{2\pi }P_{\theta }\cdot \;\sin\left(\theta \right)\;d\theta \;d\varphi$ = 1 and $\int_{0}^{\pi }\int_{0}^{2\pi }P_{\varphi }\cdot \;\sin\left(\varphi \right)\;d\theta \;d\varphi$ = 1 are the normalized power. In Equation (6), the terms $XPR\cdot \;E_{\theta i}\cdot E_{\theta j}^{*}\cdot \;P_{\theta }$ and $XPR\cdot \;E_{\varphi i}\cdot E_{\varphi j}^{*}\cdot \;P_{\varphi }$ are related to $\vec{F_{i}}\left(\theta ,\varphi \right)$, where the term $\int_{0}^{\pi }\int_{0}^{2\pi }\sin\left(\theta \right)\;d\theta \;d\varphi$ is represented by the solid angle dΩ.

The DG illustrates how well a signal is conveyed while experiencing the least amount of data loss [28], which is based on the ECC value and can be obtained by using Equation (8). The DG should be >9 dB. As ECC values decrease, the resulting DG becomes more contrasting. The ECC and DG of the proposed antenna are satisfactory.

(8) $$DG=\sqrt{1-{\left|ECC\right|}^{2}}$$

Channel losses will occur during the correlation of diversity performance. The channel losses can be estimated through the total active reflection coefficient (TARC), which can be calculated using Equation (9) [29]. TARC is the ratio of the square root of the sum of total reflected waves ($b_{i})$ to the square root of the sum of total incident waves ($a_{i})$.

(9) $$\mathrm{TARC}=\frac{\sqrt{\sum_{i=1}^{N}{\left|b_{i}\right|}^{2}}}{\sqrt{\sum_{i=1}^{N}{\left|a_{i}\right|}^{2}}}$$

For a two-port antenna system, TARC can be calculated using (10)

(10) $$\;\;\mathrm{TARC}=\Gamma_{a}^{t}=\frac{\sqrt{\left({\left|S_{11}+S_{12}e^{j\theta }\right|}^{2}\right)+\left({\left|S_{21}+S_{22}e^{j\theta }\right|}^{2}\right)}}{\sqrt{2}}\;$$

where $e^{j\theta }=\cos\theta +j\sin\theta$.

The TARC calculation is obtained by considering *θ* as 0°; $e^{j\theta }\;$= 1.

**Figure 15:**
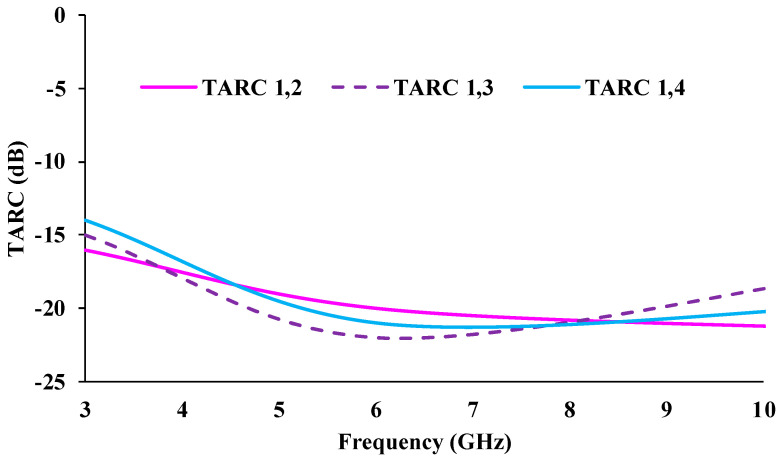
TARC plots of the MIMO antenna.

**Figure 16:**
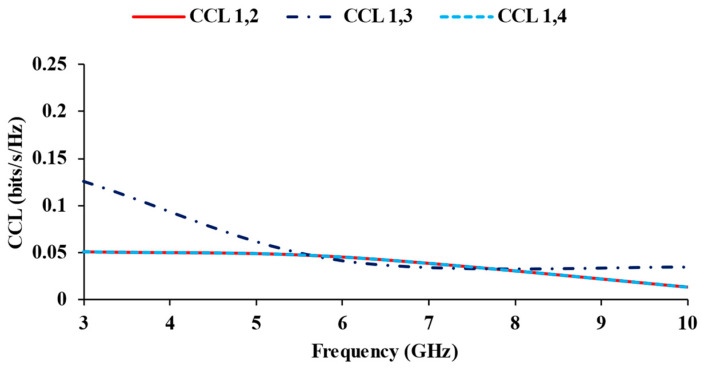
CCL plots of the MIMO antenna.

TARC should be less than −10 dB. Transmission loss is defined as channel capacity loss (CCL) in high data rate transmission [30,31], which is evaluated using Equation (11). The CCL value should not be higher than 0.4 bits/s/Hz. The calculated TARC and CCL results of the designed antenna are plotted in Figure 15 and Figure 16.

(11) $$CCL=-log_{2}{\left|\psi \right|}^{R}$$

where ${\left|\psi \right|}^{R}$ is the correlation matrix of the receiving antenna equivalent to $\left|\begin{array}{cc} \rho_{11} & \rho_{12}\\ \rho_{21} & \rho_{22} \end{array}\right|$ and

$$\begin{array}{l} \rho_{11\;}=\left(1-{\left|S_{11}\right|}^{2}-{\left|S_{12}\right|}^{2}\right),\;\rho_{12}=-(S_{11}*S_{12}+S_{21}*S_{12})\\ \rho_{21}=-(S_{22}*S_{21}+S_{12}*S_{21}),\;\rho_{22}=\left(1-{\left|S_{22}\right|}^{2}-{\left|S_{21}\right|}^{2}\right) \end{array}$$

## 5. Bending Analysis

**Figure 17:**
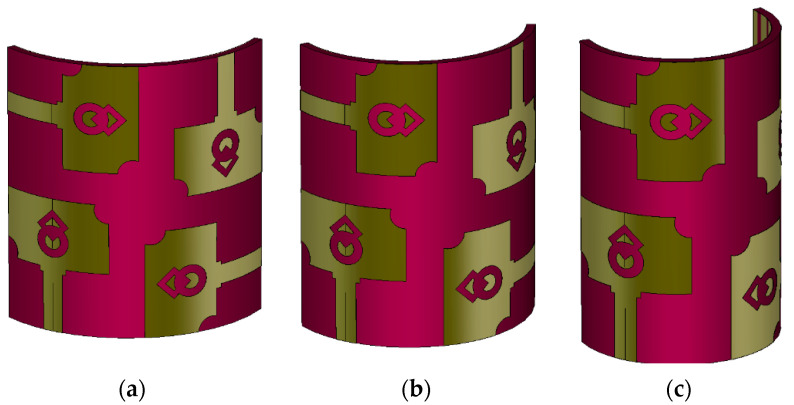
Bending analysis of the antenna at three bending radii (**a**) *BR* = 25 mm, (**b**) *BR* = 20 mm, (**c**) *BR* = 15 mm.

**Figure 18:**
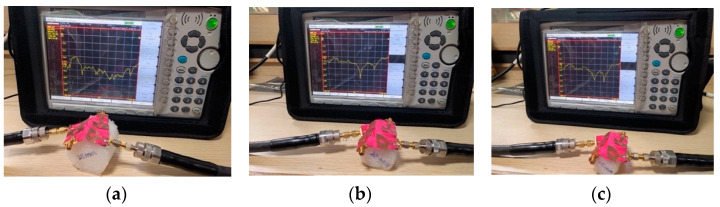
Bending analysis of the prototype antenna (**a**) *BR* = 25 mm, (**b**) *BR* = 20 mm, (**c**) *BR* = 15 mm.

**Figure 19:**
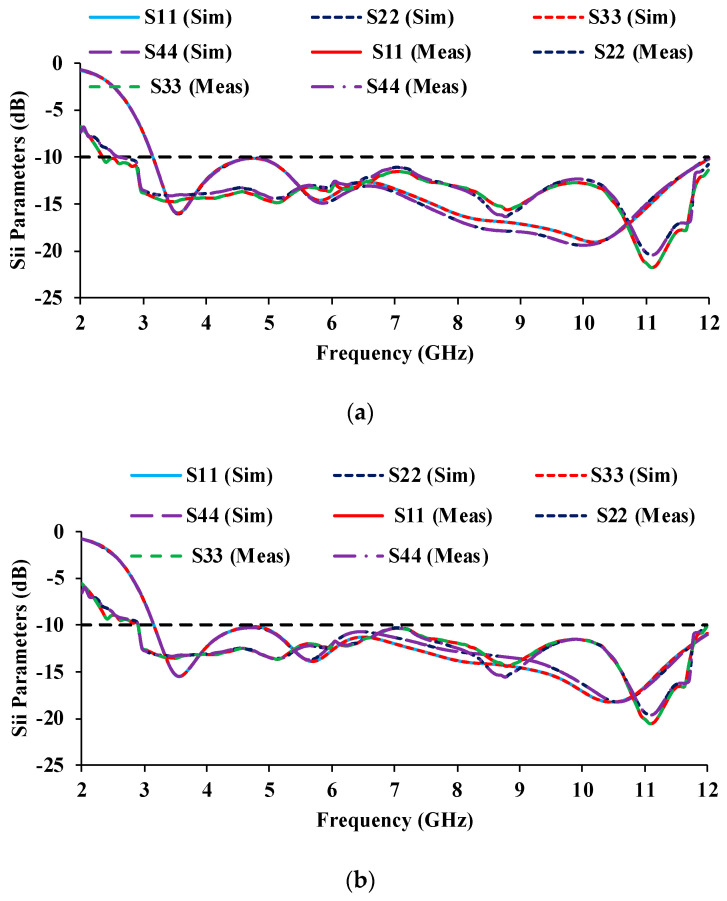

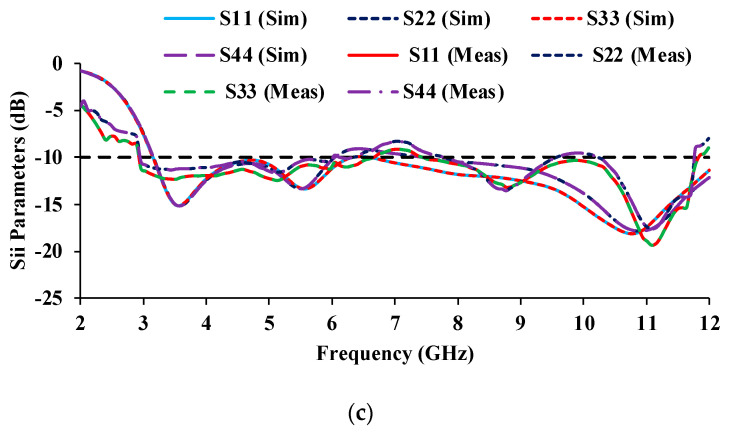
Reflection coefficients of the antenna at three bending radii (**a**) *BR* = 25 mm, (**b**) *BR* = 20 mm, (**c**) *BR* = 15 mm.

**Figure 20:**
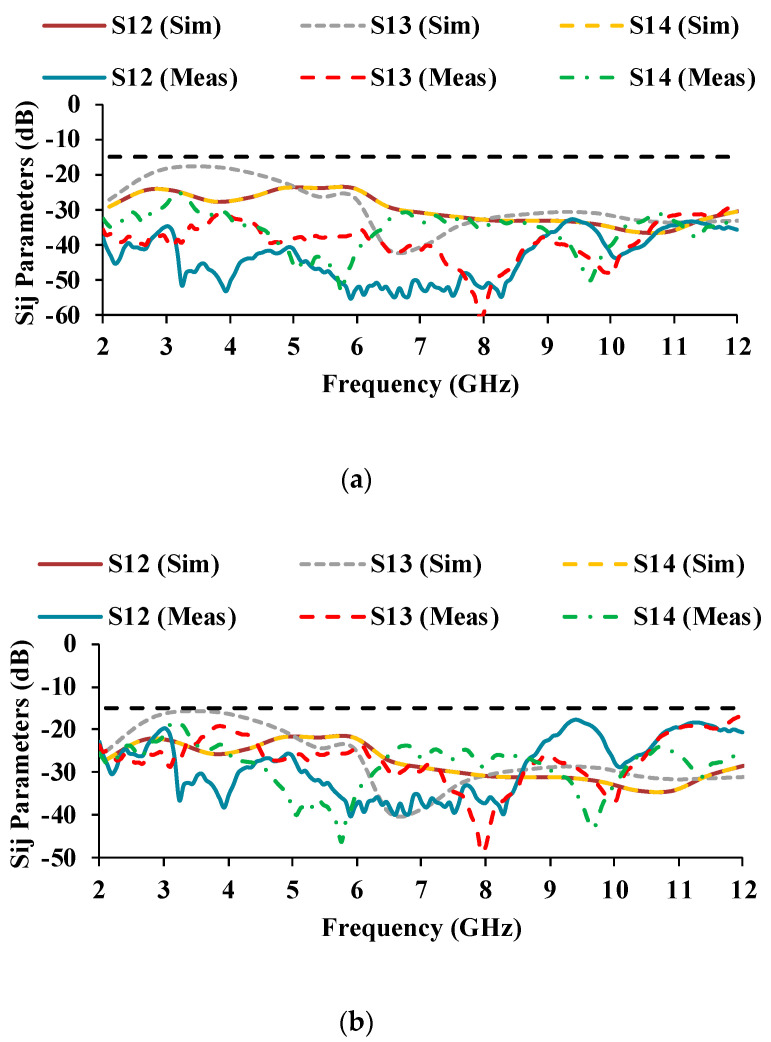

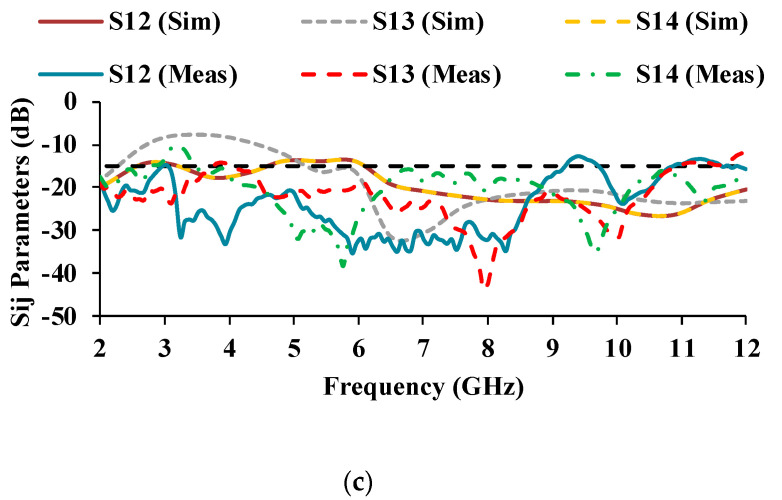
Transmission coefficients of the antenna at three bending radii (**a**) *BR* = 25 mm, (**b**) *BR* = 20 mm, (**c**) *BR* = 15 mm.

Figure 17 and Figure 18 depict the bending analysis of the antenna at three bending radii (*BR* = 25 mm, 20 mm, and 15 mm). The proposed MIMO antenna has a half-width of 25.5 mm. Therefore, 25 mm is chosen as the bending radius, and the bending performance of the antenna design is evaluated. In Figure 19 and Figure 20, the reflection coefficients and mutual coupling characteristics are plotted for three bending radii. It is observed that the proposed antenna works well when bent at 25 mm and 20 mm. The reflection coefficient values deteriorate when the antenna is bent a further 15 mm. Similarly, the mutual coupling is increased above −10 dB. Bending analysis reveals that the proposed antenna is flexible and can bend up to a 20 mm bending radius. The bending angle (*θ*) of the antenna is calculated using the following equation [32]

(12) $$\mathrm{Bending}\;\mathrm{angle}\;\left(\theta \right)=\frac{l_{y}\times 360}{r\times 2\pi }$$

where $l_{y}$ is the length of the antenna in the *y*-plane and r is the bending radius. The calculated bending angles are tabulated in Table 1.

Table 1 shows that the proposed MIMO flexible antenna can bend up to a bending angle of 146.2°, and the critical bending angle of the proposed antenna is 194.9°.

## 6. SAR Analysis

**Figure 21:**
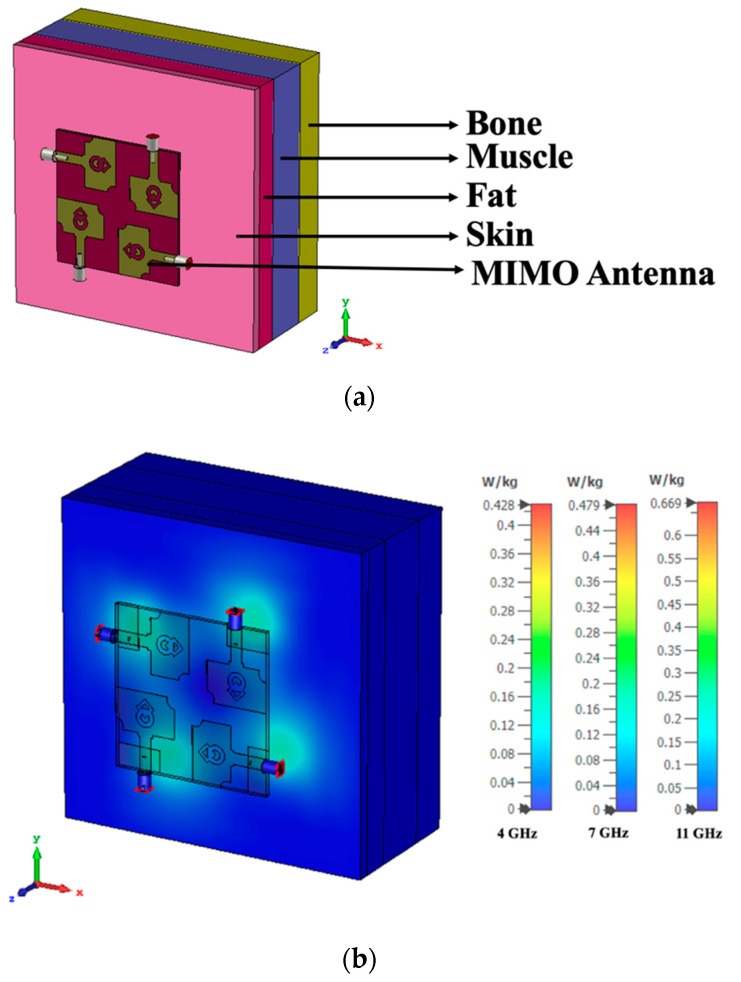

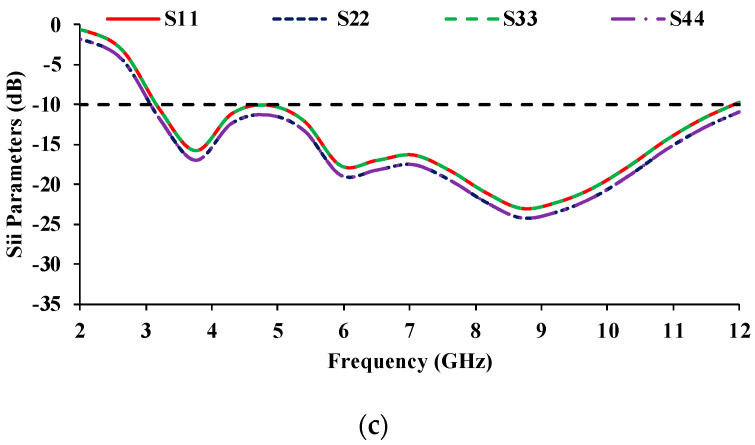
SAR analysis of the MIMO antenna (**a**) Simulated prototype, (**b**) SAR values, (**c**) Reflection coefficient curves.

In the realm of wearable technologies, a wearable antenna with lower SAR values is important [33]. The proposed antenna is designed for bio-healthcare applications. Therefore, it is essential to perform SAR analysis to determine the radiation exposure of the antenna to the human body. A rectangular human body model with human tissue layers is used to simulate the MIMO antenna, as shown in Figure 21a. The proposed antenna is simulated for 1 g of tissue by positioning it 5 mm above the quadrilateral (skin, fat, muscle, and bone) tissue model. The thickness and electrical behavior of the human body [34] tissues are listed in Table 2. The fed input power is 1 W. Figure 21b shows that the SAR results of the designed MIMO antenna are within the 1.6 W/Kg limit for all four antenna elements, making the proposed antenna suitable for bio-healthcare applications. The SAR values obtained from ports-1 and -3 are identical. Similarly, the SAR values obtained from ports-2 and -4 are identical. This is because antennas-1 and -3 are vertically oriented, whereas antennas-2 and -4 are horizontally oriented. In Figure 21c, the reflection coefficients of the MIMO antenna are plotted to test the working performance of the antenna during SAR analysis. The UWB range is evident in free space, with an impedance bandwidth of 9 GHz (3–12 GHz). Due to their similar orientations, ports-1 and -3 have similar S-parameter values, and ports-2 and -4 have similar S-parameter values.

**Figure 22:**
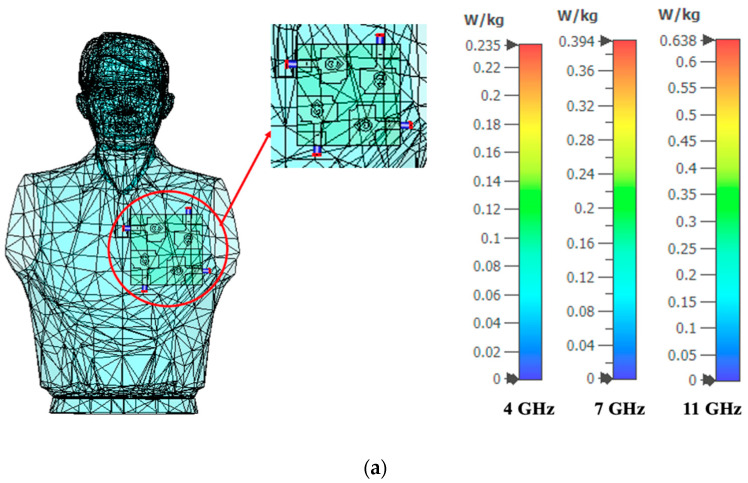

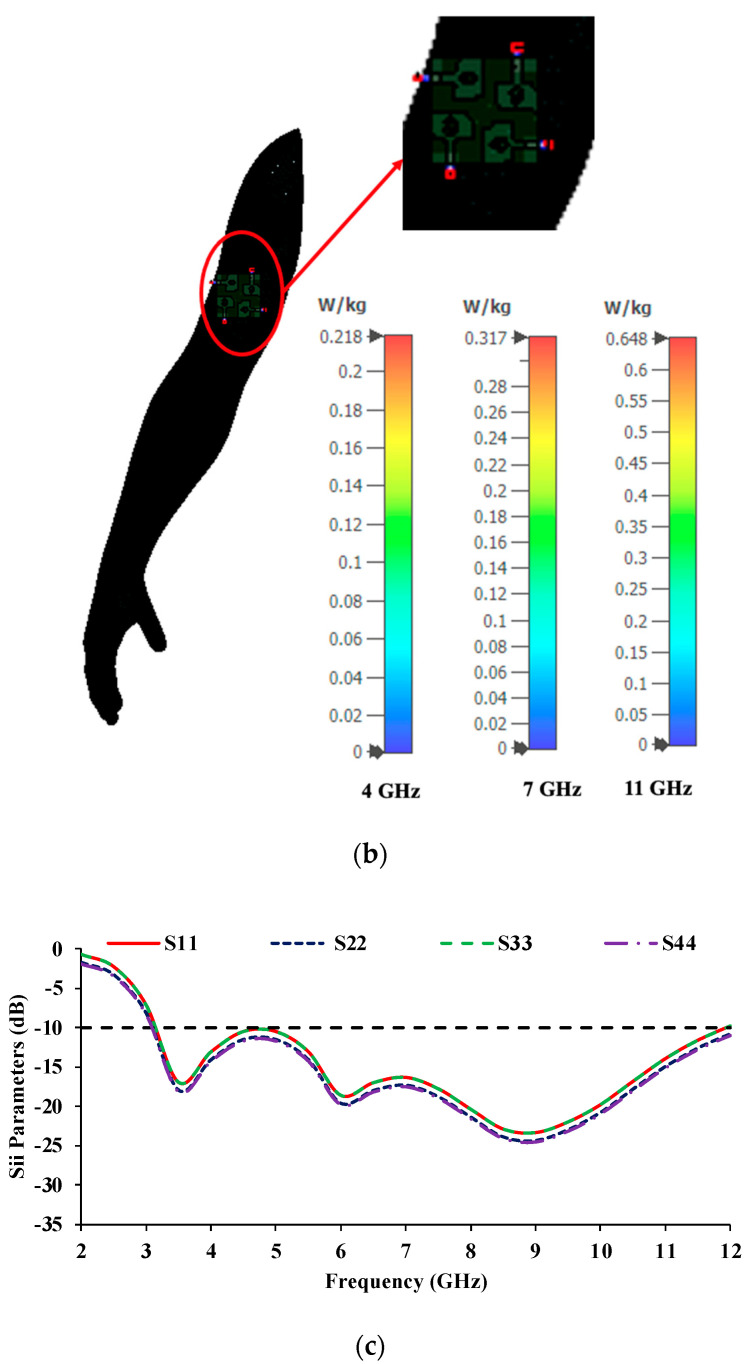
SAR analysis on an imported human body model (**a**) Chest, (**b**) Forearm, (**c**) Reflection coefficient curves.

The SAR analysis is also carried out by importing human body models from the open-source computer-aided designing (CAD) model [35]. The proposed MIMO antenna is simulated by placing it to the chest and forearm of the human body model. The SAR values of the MIMO antenna located on the chest and forearm are depicted in Figure 22a,b. The corresponding S-parameter curves of the MIMO antenna are plotted in Figure 22c, which shows an impedance bandwidth of 9 GHz (3–12 GHz).

In Figure 21, the SAR analysis is performed using a quadrilateral tissue model with the tissue thickness of skin = 2 mm, fat = 5 mm, muscle = 10 mm, and bone = 7 mm. The relative permittivity and loss tangent values of skin, fat, muscle, and bone are shown in Table 2. In contrast, the imported human body model in Figure 22 has only one layer of tissue (skin). During the SAR analysis, the antenna radiation passes through the quadrilateral tissue model with a total thickness of 24 mm with different tissue properties, which is not the case with the imported human body model. Therefore, the SAR values obtained using the quadrilateral tissue model are greater than the SAR values obtained from the imported human body model.

**Figure 23:**
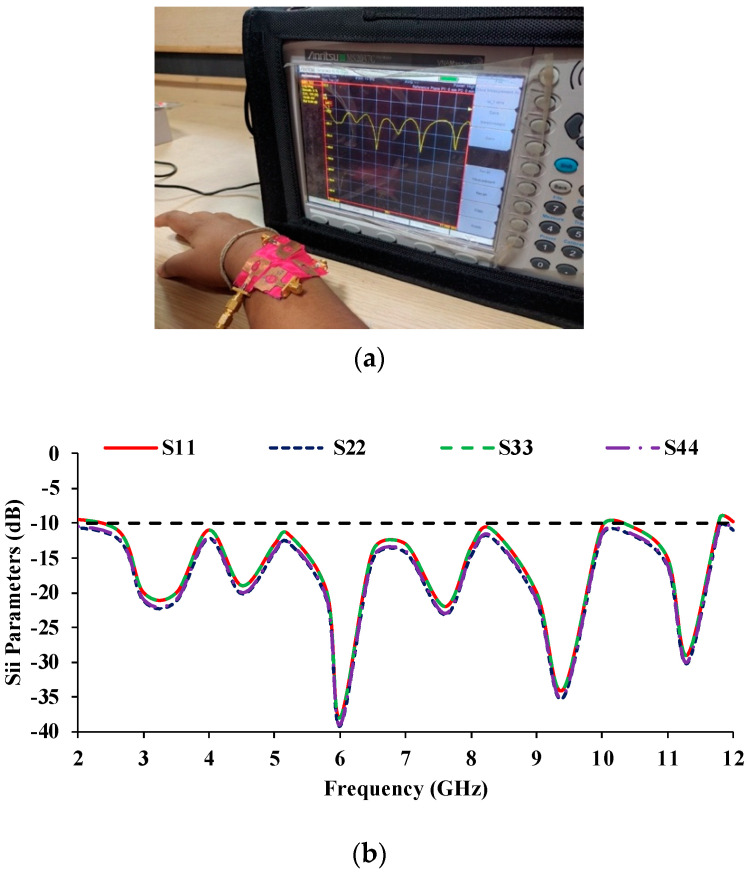
SAR analysis of the MIMO prototype antenna (**a**) VNA measurement, (**b**) Reflection coefficient curves.

The on-body performance of the proposed MIMO antenna is evaluated by placing it on a human body and measuring it with a vector network analyzer (VNA), shown in Figure 23a. Figure 23b depicts the measured reflection coefficients of the proposed MIMO antenna when it is placed on the human body. It covers the entire UWB with an impedance bandwidth of 9.2 GHz (2.6–11.8 GHz), making it suitable for wearable and bio-healthcare applications.

**Figure 24:**
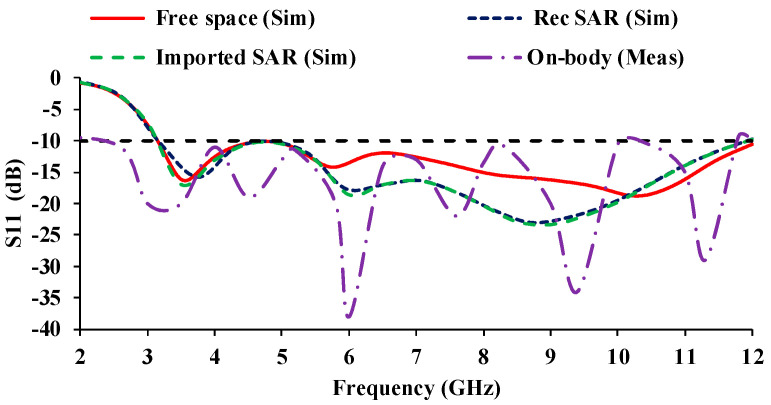
Reflection coefficient of SAR analysis of a MIMO prototype.

Figure 24 compares the reflection coefficient curves obtained from different SAR calculations. The results show that there is no discernible change in the reflection coefficient curves (in both simulation and measurement) when the proposed antenna is subjected to SAR performance. In all of the cases depicted in Figure 24, the proposed antenna covers the entire UWB range.

Table 3 compares the performance of the proposed antenna with the previously published antenna designs.

- The proposed antenna has a smaller unit cell size than [36,37,38,39,40,41,42,46].
- In comparison to the other antenna designs, the proposed four-port MIMO antenna size is relatively small.
- The designed antenna has a broader impedance bandwidth of 8.9 GHz than the [36,37,38,39,40,42,44,45,46] antennas.
- The developed antenna has a peak gain of 4.62 dBi when compared to [38,39,42,45,46].
- Unlike [38,39,41,42,43], the diversity gain of the designed antenna is more than 9.99 dB.

The following are the main highlights of the proposed work:

- The proposed structure is a compact MIMO antenna composed of four resonators with dimensions of 0.52$\lambda_{0}$ × 0.52$\lambda_{0}$ × 0.015$\lambda_{0}$, where $\lambda_{0}$ represents the lowest operating frequency.
- The proposed antenna is made of cost-effective textile material, and it is constructed with simple-to-fabricate structures.
- The proposed antenna is lightweight and easy to integrate into the human body.
- The proposed antenna fabrication process is simple, easy, and inexpensive, and it allows for easy integration into clothing.
- The flexibility of the proposed antenna is evaluated using bending analysis at different bending radii.
- The proposed antenna has high durability, resistance to wrinkles, and resistance to strain due to the use of the substrate polyester.
- The rectangular human body model and the imported human body CAD model showed low SAR values.
- The proposed MIMO antenna has two polarization vectors (vertical and horizontal) and exhibits polarization diversity.

## 7. Conclusions

A smart textile UWB MIMO antenna design is proposed for bio-healthcare applications. The unit cell is developed into a four-port MIMO antenna with each antenna element facing orthogonally to the other. Isolation >17 dB is obtained across the entire UWB band, and the calculated diversity measures of the designed antenna are satisfactory. The obtained SAR values are significantly lower than 1.6 Watt/Kg, making the antenna suitable for wearable, smart textiles, location tracking, patient monitoring, and sports applications. The radiation exposure can be further reduced by using periodic structures such as EBG, frequency selective surfaces, and so on.

## Figures and Tables

**Table 1:** Bending angles at different bending radii.

Bending Radius (*BR*)	Bending Angle (°)
25 mm	116.94
20 mm	146.2
15 mm	194.9

**Table 2:** Electrical properties of human body tissues.

Human Body Tissue Layers	Frequency (GHz)	Relative Permittivity (*ε_r_*)	tan *δ*	Thickness of Tissue Layers (mm)
Skin	4	36.6	0.281	2
	7	34.1	0.36	
	10	31.3	0.47	
Fat	4	5.12	0.14	5
	7	4.85	0.19	
	10	4.6	0.24	
Muscle	4	50.8	0.23	10
	7	46.9	0.33	
	10	42.8	0.45	
Bone	4	10.5	0.16	7
	7	9.17	0.183	
	10	8.12	0.21	

**Table 3:** Comparison of the proposed antenna with the previously published antenna designs.

Ref.	Substrate	Unit Cell Area	MIMO Area	No. of Ports	Bandwidth (GHz)	Peak Gain (dBi)	DG (dB)
[36]	Denim	36 × 29	---	1	3–11	7.2	---
[37]	Jeans cotton	65 × 60	---	1	2.19–3	---	---
[38]	Jeans	16 × 28	40 × 86	2	2.42–2.47	3	>9.5
[39]	Felt	37 × 34	37 × 76	2	2–6.23	2.88	9.95
[40]	Felt	36 × 27	36 × 54	2	1.1–8.6	7.5	---
[41]	Felt	30.5 × 20	32.5 × 42	2	3.6–13	5.7	>9.96
[42]	Felt	47.2 × 31	132.8 × 70	2	3.53–7.1	1.878, 4.027	>9.975
[43]	Jeans	20 × 16	20 × 32	2	3.38–12.78	---	9.99
[44]	Jeans	26 × 12	26 × 24	2	4.9–6	5.1	10
[45]	Kapton	22 × 15	22 × 31	2	3.43–10.1	3.5	---
[46]	FR-4	31 × 22	31 × 44	2	2.28–2.47 3.4–3.62 4.57–6.75	1.3 2.9 4.3	9.998 9.999 9.998
Prop.	Polyester	25 × 20	51 × 51	4	3.1–12	4.62	>9.99

## Data Availability

Not applicable.
